# Efficacy of one-stage cartilage repair using allogeneic mesenchymal stromal cells and autologous chondron transplantation (IMPACT) compared to nonsurgical treatment for focal articular cartilage lesions of the knee: study protocol for a crossover randomized controlled trial

**DOI:** 10.1186/s13063-020-04771-8

**Published:** 2020-10-09

**Authors:** J. V. Korpershoek, L. A. Vonk, E. C. Kester, L. B. Creemers, T. S. de Windt, M. M. A. Kip, D. B. F. Saris, R. J. H. Custers

**Affiliations:** 1 Department of Orthopaedics, University Medical Center Utrecht, Heidelberglaan 100, 3584CX Utrecht, The Netherlands; 2 Present Address: CO.DON AG, Warthestraße 21, D-14513 Teltow, Germany; 3 Department of Health Technology and Services Research, Technical Medical Centre, University of Twente, Technohal, Hallenweg 5, 7522 NH Enschede, The Netherlands; 4 Department of Orthopedic Surgery and Sports Medicine, Mayo Clinic, 200 First Street SW, Rochester, MN 55905 USA

**Keywords:** Articular cartilage defect, Chondrocytes, Chondrons, Mesenchymal stromal cells (MSC), Surgery, Knee, Randomized controlled trial (RCT), Crossover design, Advanced therapy medicinal product (ATMP)

## Abstract

**Background:**

Articular cartilage defects in the knee have poor intrinsic healing capacity and may lead to functional disability and osteoarthritis (OA). “Instant MSC Product accompanying Autologous Chondron Transplantation” (IMPACT) combines rapidly isolated recycled autologous chondrons with allogeneic MSCs in a one-stage surgery. IMPACT was successfully executed in a first-in-man investigator-driven phase I/II clinical trial in 35 patients. The purpose of this study is to compare the efficacy of IMPACT to nonsurgical treatment for the treatment of large (2–8 cm^2^) articular cartilage defects in the knee.

**Methods:**

Sixty patients will be randomized to receive nonsurgical care or IMPACT. After 9 months of nonsurgical care, patients in the control group are allowed to receive IMPACT surgery. The Knee Injury and Osteoarthritis Outcome Score (KOOS), pain (numeric rating scale, NRS), and EuroQol five dimensions five levels (EQ5D-5 L) will be used to compare outcomes at baseline and 3, 6, 9, 12, and 18 months after inclusion. Cartilage formation will be assessed at baseline, and 6 and 18 months after inclusion using MRI. An independent rheumatologist will monitor the onset of a potential inflammatory response. (Severe) adverse events will be recorded. Lastly, the difference between IMPACT and nonsurgical care in terms of societal costs will be assessed by monitoring healthcare resource use and productivity losses during the study period. A health economic model will be developed to estimate the incremental cost-effectiveness ratio of IMPACT vs. nonsurgical treatment in terms of costs per quality adjusted life year over a 5-year time horizon.

**Discussion:**

This study is designed to evaluate the efficacy of IMPACT compared to nonsurgical care. Additionally, safety of IMPACT will be assessed in 30 to 60 patients. Lastly, this study will evaluate the cost-effectiveness of IMPACT compared to nonsurgical care.

**Trial registration:**

NL67161.000.18 [Registry ID: CCMO]

2018#003470#27 [EU-CTR; registered on 26 March 2019]

NCT04236739 [ClinicalTrials.gov] [registered after start of inclusion; 22 January 2020]

## Administrative information

Note: the numbers in curly brackets in this protocol refer to SPIRIT checklist item numbers. The order of the items has been modified to group similar items (see http://www.equator-network.org/reporting-guidelines/spirit-2013-statement-defining-standard-protocol-items-for-clinical-trials/).

**Table:** 

Title {1}	Efficacy of one-stage cartilage repair using allogeneic mesenchymal stromal cells and autologous chondron transplantation (IMPACT) compared to nonsurgical treatment for focal articular cartilage lesions of the knee: study protocol for a crossover randomized controlled trial
Trial registration {2a and 2b}.	2018-003470-27 [EU Clinical Trials Register (EU-CTR)/Eudra CT] https://www.clinicaltrialsregister.eu/ctr-search/search?query=2018-003470-27 [registered on 26-03-2019] NCT04236739 [ClinicalTrials.gov] [registered after start inclusion; 22-01-2020] https://clinicaltrials.gov/ct2/show/NCT04236739 NL67161.000.18 [Registry ID: CCMO] [registered on 24-09-2018]
Protocol version {3}	Version 5 of 19-9-2019
Funding {4}	This research is funded by ZonMw (The Netherlands Organization for Health Research and Development) and the strategic theme ‘Regenerative Medicine & Stem Cells’ of the University Medical Center Utrecht
Author details {5a}	J.V. Korpershoek: University Medical Center Utrecht, the Netherlands L.A. Vonk: University Medical Center Utrecht, the Netherlands (current address: CO.DON AG, Teltow, Germany) E.C. Kester: University Medical Center Utrecht, the Netherlands L.B. Creemers: University Medical Center Utrecht, the Netherlands T.S. de Windt: University Medical Center Utrecht, the Netherlands M.M.A. Kip: technical medical center, University of Twente, the Netherlands D.B.F. Saris: University Medical Center Utrecht, the Netherlands; Mayo Clinic, Rochester, Minnesota, USA R.J.H. Custers: University Medical Center Utrecht, the Netherlands
Name and contact information for the trial sponsor {5b}	Investigator initiated clinical trial; R.J.H. Custers (Principal Investigator) r.j.h.custers@umcutrecht.nl
Role of sponsor {5c}	This is an investigator initiated clinical trial. Therefore, the funders played no role in the design of the study and collection, analysis, and interpretation of data and in writing the manuscript.

## Introduction

### Background and rationale {6a}

Cartilage defects are common knee injuries that lead to a deterioration of sports performance, increased work leave, and limitations in daily activities. Cartilage defects may eventually lead to osteoarthritis (OA) due to the limited healing capacity of cartilage [1, 2]. Treatment aims at obtaining a pain-free joint function by achieving structural tissue repair. Small cartilage defects are successfully treated using microfracture, but treatment of large defects (2–8 cm^2^) requires more advanced techniques. The application of fresh allografts for large or deep defects is limited by the high costs and poor availability [3]. Synthetic implants are easy to use and short-term results look promising, but the quality of the repair tissue is poor [4, 5]. Autologous chondrocyte implantation (ACI) is a two-stage treatment, in which a biopsy of healthy cartilage from a non-weight bearing location in the knee is taken during an initial knee arthroscopy. Approximately 180,000–455,000 chondrocytes are isolated from such a biopsy [6], while millions of cells per milliliters of defect filling are needed [7, 8]. In order to obtain sufficient chondrocytes to repair the cartilage defect, a period of cell expansion is required [6]. After approximately 4–13 weeks, the cultured autologous chondrocytes are re-implanted into the cartilage defect in a second surgical procedure. Although ACI procedures showed good mid-term and long-term results [9, 10], chondrocyte expansion leads to a decrease in type II collagen and increase in type I collagen gene expression, which are both signs of dedifferentiation [11, 12]. Furthermore, ACI is a costly procedure due to the requirement of cell culture [13] and it has been unavailable in many European countries after different products have been withdrawn from the European market [14, 15]. Due to this limited availability, nonsurgical care consisting of physiotherapy and pain medication remains the treatment of choice.

Both in vitro and in vivo cartilage formation has been shown to improve by direct contact between multipotent mesenchymal stromal cells (MSCs) and articular chondrocytes [16–20]. This stimulatory effect on cartilage matrix formation further increases when MSCs are combined with chondrons (chondrocytes with their pericellular matrix) [19]. Using this combination of cells allows for a one-stage application, as autologous cells can be used without expansion. “Instant MSC Product accompanying Autologous Chondron Transplantation” (IMPACT) combines 10% autologous chondrons with 90% allogeneic MSCs in a single surgery for the treatment of cartilage defects. Compared to the two-stage ACI procedure, IMPACT decreases the patient burden and significantly reduces the costs of treatment [13]. Safety, feasibility, initial efficacy, and structural tissue repair of IMPACT was shown in a cohort of 35 patients with cartilage lesions (3.2 ± 0.7 cm) (NCT02037204) [21, 22].

### Objectives {7}

The current phase III randomized controlled trial explores the efficacy of IMPACT compared to nonsurgical care in 60 patients with large (2–8 cm^2^) articular cartilage defects of the knee. We allow patients in the nonsurgical group to cross-over to the IMPACT group after 9 months of nonsurgical care. Follow-up will be at least 18 months after IMPACT. The primary objective is to compare the Knee Injury and Osteoarthritis Outcome Score (KOOS) at 3, 6, and 9 months follow-up. The secondary objective of this study is to examine morphology and proteoglycan content of repair tissue 6 and 18 months after treatment. Safety endpoints will be determined by the number of (treatment-related) adverse events. In addition, the incremental cost-effectiveness ratio (ICER) of IMPACT vs. nonsurgical care and vs. delayed surgical intervention will be calculated. More specifically, the effect of IMPACT compared with nonsurgical care and delayed surgical intervention in terms of healthcare resource use, productivity losses, and accompanying costs during the study period will be determined and extrapolated to a 5-year time horizon.

### Additional consent provisions for collection and use of participant data and biological specimens {26b}

A subgroup of 15 patients will be asked to participate in an optional study, in which the structure and composition (glycosaminoglycan content) of the regenerated tissue are studied using high-resolution imaging. These patients will undergo additional MRI-scans using a 7-Tesla MRI-scanner at baseline, 6, and 18 months. Regardless of treatment allocation, patients can volunteer for this part of the trial by indication on the informed consent form.

### Trial design {8}

In this phase III randomized controlled clinical trial, IMPACT is compared to nonsurgical treatment. The patient allocation ratio is 1:1. Patients in the nonsurgical group are allowed to cross over to the treatment group after 9 months follow-up.

## Methods: participants, interventions, and outcomes

### Study setting {9}

The IMPACT-trial will be performed in a tertiary referral hospital (University Medical Center Utrecht (UMC Utrecht)) in the Netherlands that is specialized in the treatment of cartilage defects of the knee. Patients are recruited at the Mobility Clinic, which includes the outpatient clinic of orthopedics, sports medicine, and rheumatology and is part of the UMC Utrecht. Patients are considered for inclusion if they meet the criteria as defined below.

### Eligibility criteria {10}

#### Primary inclusion criteria (at the outpatient clinic)

Patients must meet the following criteria to be eligible for the study:

- The patient provides written informed consent, is able to understand the content of the study, understands the requirements for follow-up visits, and is willing to complete the questionnaires and provide the required information at follow-up visits.
- Symptomatic articular cartilage defect of the knee (femoral condyles or trochlea) 2–8 cm^2^.
- Age ≥ 18 and ≤ 45 years old.

#### Primary exclusion criteria (at the outpatient clinic)

If the patients meet any of the following criteria at the screening visit, they will not be eligible for the study:

- Malalignment of > 5° (correctional osteotomy is allowed during the trial).
- (History of) OA, defined as Kellgren-Lawrence grade ≥ 3 as determined from appropriate radiography.
- Joint instability (ligament reconstruction is allowed during the trial).
- Concomitant inflammatory disease that affects the joint (rheumatoid arthritis, metabolic bone disease, psoriasis, gout, symptomatic chondrocalcinosis).
- (History of) Septic arthritis.
- (History of) Total meniscectomy in the target knee joint.
- Any surgery in the index knee joint 6 months prior to study inclusion.
- Risk groups for MRI due to the magnetic field such as patients with pacemakers, nerve stimulators, metal particles, stents, clips or implants, (possible) pregnancy, or breastfeeding.

Definitive eligibility is assessed during surgery based on the criteria below.

#### Definitive inclusion criteria (during surgery)

Patients must meet the following criteria to be eligible for IMPACT:

- Modified Outerbridge Grade III or IV isolated cartilage lesion of the knee.
- A post-debridement size of the cartilage lesion ≥ 2 cm^2^ and ≤ 8 cm^2^.
- At least 50% of functional meniscus remaining. Meniscal repair or resection is allowed during the IMPACT surgery provided that the surgeon is able to confirm that at least 50% of functional meniscus remains.
- Stable knee ligaments (i.e., anterior and posterior cruciate ligaments).

#### Definitive exclusion criteria (during surgery)

Patients who meet any of the following criteria at surgery will not be eligible for IMPACT:

- Patients with lesions > 8 cm^2^.
- Patients with osteoarthritic lesions Kellgren-Lawrence grade ≥ 3 not diagnosed before surgery.

### Who will take informed consent? {26a}

Patients with an MRI- or previous arthroscopically confirmed isolated articular cartilage lesion will be screened for eligibility to participate in this study based on the abovementioned criteria. After the patient has been assessed as eligible by the treating orthopedic surgeon, he/she will receive initial study information. After at least 2 weeks of reflection, patients are invited to meet with the research physician to discuss any remaining questions and sign the informed consent.

## Interventions

### Intervention description {11a}

IMPACT is a one-stage cell-based regenerative therapy for isolated articular cartilage lesions. The investigational product consists of 10% autologous chondrons recycled from the debrided defect tissue and 90% allogeneic MSCs in Tisseel® tissue glue (Baxter B.V, Utrecht, the Netherlands) which will act as a cell carrier for implantation. The MSCs are obtained from the bone marrow of healthy non-HLA matched donors in the GMP-licensed Cell Therapy Facility (Department of Clinical Pharmacy, University Medical Center Utrecht) and cultured and characterized as described previously [22]. To summarize, the bone marrow aspirate is density-separated and MSCs are isolated using plastic adherence. The MSCs are expanded up to passage three after which they are cryopreserved. MSCs are characterized by the expression of CD73, CD105, and CD90 and the absence of expression of CD45, and CD3.

IMPACT surgery [21, 22] consists of a mini-arthrotomy, during which the cartilage defect is debrided and stable borders are created. The debrided cartilage tissue is transported to the Cell Therapy Facility, where chondrons are isolated from the tissue using a rapid digestion protocol; minced cartilage is digested in 40 min in Liberase MNP-S GMP Grade (Roche, Mannheim, Germany). The autologous chondron-suspension is run over a 100-μm strainer (Corning Inc., New York, USA) to remove the undigested cartilage matrix. The chondrons are washed twice to remove the enzyme and counted using 3% acetic acid with methylene blue (STEMCELL Technologies Germany GmbH, Köln, Germany). Allogeneic cryopreserved MSC are thawed and the chondrons and MSCs are mixed at a 10:90 ratio in the fibrinogen component of Tisseel®. The fibrinogen and thrombin component of Tisseel® are mixed upon application, which causes the product to gelate. Two million cells per milliliter are implanted in the defect. The rehabilitation protocol we use is equal to that after ACI. Briefly, during rehabilitation, patients are allowed 10% weight-bearing the first 3 weeks, after which the load is increased gradually up to 50% at 6 weeks and 100% at 8 to 12 weeks. From 5 months onward, the rehabilitation protocol aims at improving coordination, increasing muscle strength, and becoming functional in moderately intensive activities. Patients can return to low-impact sports after 9 months and to high-impact sports at the earliest after 12 months. Patients treated for cartilage defects in the trochlea are allowed weight-bearing in the first 6 weeks after surgery but will use an extension brace in order to limit the flexion while weight-bearing.

### Explanation for the choice of comparators {6b}

The control group receives standard care, which is nonsurgical. Due to the varying availability of ACI in the Netherlands, this is the comparator of choice for cartilage defects of 2–8 cm^2^. The control group is allowed the option to take pain medication at their own discretion as well as physical therapy by their own physical therapist.

### Criteria for discontinuing or modifying allocated interventions {11b}

Patients can leave the study at any time for any reason if they wish to do so without any consequences. The patient’s participation in this study can also be ended by the investigator if the patient is uncooperative and/or does not attend study visits. The patient data that have been collected up to that moment will be included in the analysis. In case too many data are missing (e.g., missing baseline or all of the follow-up patient-reported outcome measures (PROMs), study visits, or MRI-scans), the patient will be replaced by a new patient. This study will be prematurely ended in case of any abundance in adverse events or procedure/compound-related complications or if the independent rheumatologist advises this termination. Criteria for study termination include any suspected unexpected serious adverse reaction (SUSAR) or serious adverse event (SAE) based on an allergic reaction and clear allergic or iatrogenic effects in two or more patients including patients which report back to the hospital with serious iatrogenic complaints. In case of premature ending, all included patients will be informed by their treating orthopedic surgeon. In case of illness, patients will be asked to contact the primary investigator. Patients that are discovered during surgery not to meet the criteria will not receive IMPACT and will be treated according to the standard of care, based on the findings during surgery. These patients will be removed from the study. Patient data included up to that moment will be included in the analysis.

#### Out of Specification product

An Out of Specification (OOS)-product is a product that cannot be made according to the criteria, for example when an insufficient number of chondrons is isolated. In this case, a risk-benefit assessment of implantation of the OOS-product will be done by the treating orthopedic surgeon and qualified person. They will consider alternative treatment options for the patient, whether manufacturing can be (partly) repeated, and the ratio of the cells restored. The OOS-product will contain a higher percentage of MSCs in order to compensate for the missing chondrons, and two million cells per milliliter will be implanted. Patients that receive an OOS-product will not be included in efficacy analyses, but AE, SAE, and SUSARs will still be reported and the patient will be included in safety and cost-effectiveness analyses. Patients will be informed of this OOS-procedure both during the screening visit and through the patient information letter, before signing the informed consent form. The researchers will report implantation of an OOS-product to the Central Committee on Research Involving Human Subjects (CCMO) and Health and Youth Care Inspectorate (IGJ), according to the CCMO guidelines.

#### Treatment algorithm in case of foreign body response

If there is suspicion of an acute (within 48 h) foreign body response after surgery, a consultation by an independent rheumatologist will be requested immediately. Depending on the severity of the reaction, initial treatment will consist of NSAIDs, anti-histamines, or immunosuppressants. If no improvement occurs within 48 h, treating specialists will consider a diagnostic knee aspiration (in case of signs of infection), anaphylaxis protocol, or debridement and lavage. In case of a late immunological response (after 72 h), infection will be excluded as a cause of the reaction, prior to starting the algorithm as mentioned above.

### Strategies to improve adherence to interventions {11c}

The nonsurgical protocol consists of physiotherapy and/or pain medication and can be adjusted to the individual patient’s needs. The adherence to this protocol will be high as it does not consist of strict guidelines. Adherence to the rehabilitation protocol after IMPACT will be monitored by the specialized physiotherapists in our center. They are in close contact with the treating physiotherapists and monitor progression during study visits.

### Relevant concomitant care permitted or prohibited during the trial {11d}

Concomitant surgery such as anterior cruciate ligament reconstruction or alignment correction is permitted during the trial but will be registered. Injections into the index knee are not permitted 6 months pre- and 12 months postoperatively.

### Provisions for post-trial care {30}

The sponsor has insurance, which is in accordance with the legal requirements in the Netherlands (Article 7 Medical Research Involving Human Subjects Act (WMO)). This insurance provides coverage for damage to research subjects through injury or death caused by any activities of the study. The insurance applies to the damage that becomes apparent during the study or within 4 years after the end of the study.

### Outcomes {12}

The primary outcome is the comparison in total KOOS between patients with cartilage defects that are treated with IMPACT and patients treated with standard care (nonsurgical treatment) until 9 months after randomization. Total KOOS is an average of the scores in the five subscales of KOOS [23]. Other outcomes of interest are outcomes in the five subscales of KOOS, pain (numeric rating scale, NRS), general health (EuroQol five dimensions five levels, EQ5D-5 L), and structural repair (MRI). After 9 months, patients in the nonsurgical group are allowed to undergo IMPACT surgery, this will be regarded as failed nonsurgical treatment, and the time of crossover will be recorded. Change from baseline assessed with the KOOS of the total group of patients treated with IMPACT at 3, 6, 9, 12 and 18 months after treatment will be evaluated (per protocol). The potential effect of time until surgical treatment will be assessed. Clinical safety will be determined by active tracing of the adverse event rate observed after IMPACT and nonsurgical therapy. Additionally, emergence of an immune response will be assessed by screening for anti-HLA antibodies in peripheral blood preoperatively and 4 weeks postoperatively. The HLA phenotype of MSC donors will be compared to newly formed anti-HLA antibodies. Societal costs will be assessed by monitoring the costs related to the IMPACT procedure and the accompanying rehabilitation period, as well as costs related to nonsurgical (or delayed) treatment. In this analysis, two scenarios will be compared: (I) IMPACT vs. nonsurgical treatment and (II) IMPACT vs. delayed surgical treatment. In the first scenario, it is assumed that patients randomized to nonsurgical treatment will not undergo IMPACT in the next 5 years, whereas the second scenario includes patients who were randomized to nonsurgical care and opt for IMPACT after 9 months follow-up. In both scenarios, costs of other healthcare-related resource use (including physiotherapy, home care, medication use, and costs related to adverse events), as well as costs attributable to health-related work leave (i.e., productivity losses), will be collected over a follow-up period of (at least) 9 months. For nonsurgical treatment, health outcomes (from the EQ-5D-5L) and costs within the first 9 months will be extrapolated to calculate costs/QALY over a 5-year time horizon. Similarly, health outcomes and costs for (delayed) IMPACT will also be extrapolated to a five-year time horizon, using results from the completed phase I/II study (unpublished results).

### Participant timeline {13}

Table 1 shows the participant timeline.

**Table 1:** Participant timeline

**Outpatient Clinic:** Screening (MRI) Study information provided in writing	
**Baseline visit:** Informed consent Baseline study MRI Randomization Baseline PROMs	
Group A: IMPACT	Group B: Control (nonsurgical)
**Preoperative screening** Anti-HLA antibodies	**3 months after inclusion** PROMs Check-up study physician (by telephone)
**Surgery/1 day postoperative** Blood chemistry (CRP, ESR, Leucocytes) Visit physical therapist Check-up rheumatologist Check-up study physician	**6 months after inclusion** PROMs Check-up study physician MRI
**1 week postoperative** Blood chemistry (CRP, ESR, Leucocytes) Check-up rheumatologist Check-up study physician	**9 months after inclusion** PROMs
**4 weeks postoperative** Blood chemistry (CRP, ESR, Leucocytes, anti-HLA antibodies) Check-up rheumatologist Check-up study physician	In case of cross-over to IMPACT: follow group A In case nonsurgical treatment is continued: **12 months after inclusion:** PROMs
**3 months postoperative** Check-up study physician (by telephone) PROMs	**18 months after inclusion:** PROMs MRI
**6 months postoperative** Check-up study physician PROMs MRI	
**9 months postoperative** PROMs	
**12 months postoperative** Check-up study physician PROMs	
**18 months postoperative** Check-up study physician PROMs MRI	

*CRP* C-reactive protein, *ESR* erythrocyte sedimentation rat, *HLA* human leukocyte antigens, *IMPACT* Instant MSC Product accompanying Autologous Chondron Transplantation, *MRI* magnetic resonance imaging; *PROMs* patient-reported outcome measures

### Sample size {14}

The sample size was calculated for the primary objective (treatment effect up to 9 months postoperatively) based on the Hotelling-Lawley trace [24, 25]. Based on a standard deviation of 15 [23], correlation of the repeated measures of 0.7 (data from our phase I trial [21]), and with a power of 0.8 and alpha of 0.05, a minimum of 44 patients should be included to detect a minimal clinical relevant treatment effect of 10 for KOOS [23]. To account for potential loss to follow-up, and uncertainties in the correlation pattern and standard deviation for nonsurgical treatment, this was rounded up to 60 patients in total.

### Recruitment {15}

Patients will be recruited at the Mobility Clinic (which includes the outpatient clinic of the department of Orthopedics) of the University Medical Center Utrecht. We carry out over 250 surgical procedures for cartilage defects in our center annually.

## Assignment of interventions: allocation

### Sequence generation {16a}

Patients will be randomized into variable block sizes of two and four, stratified by defect size (< 4 or ≥ 4 cm^2^), using Castor EDC [26].

### Concealment mechanism {16b}

Allocation is not concealed and will be revealed to both the patient and the researcher upon randomization.

### Implementation {16c}

After signing the informed consent forms, the researchers will use Castor EDC [26] to allocate the patient to one of the study arms. The study group will be revealed at the same time to both the patient and researcher.

## Assignment of interventions: blinding

### Who will be blinded {17a}

Patients, researchers, and surgeons will not be blinded, since this is impossible due to the major difference between the two groups (surgical versus nonsurgical).

### Procedure for unblinding if needed {17b}

The trial design is open label, therefore there is no unblinding procedure.

## Data collection and management

### Plans for assessment and collection of outcomes {18a}

Data will be derived from electronic patient records and collected with an electronic Case Report Form (eCRF) using Castor EDC (Good Clinical Practice (GCP) Compliant) [26]. Patients will use an online survey (OnlinePROMS, InterActive Studios, Rosmalen, the Netherlands) to answer questionnaires. Laboratory tests are performed by the central diagnostic laboratory and MRIs will be made at the Department of Radiology of the UMC Utrecht. All radiographic data acquired during the study will be anonymized and saved in a study folder on our protected research server. Only the study team has access to this specific study folder. For the cost-effectiveness analysis, the resources required for the different procedures (i.e., IMPACT or nonsurgical treatment), as well as for the IMPACT product that is used (materials, operation theater, etc.), and the duration of the accompanying hospitalization will be derived from the electronic patient records. In addition, the results of the iMTA Medical Consumption Questionnaire will be used to collect data on resource use (including physiotherapist visits, home care, and medication use) and the results of the iMTA Productivity cost questionnaire will be used to collect data on productivity losses [27]. All resource use will be multiplied with cost prizes, which will be obtained from the Dutch Healthcare Authority [28], from UMC Utrecht hospital tariffs or from the Dutch manual for performing health economic evaluations [29], to calculate total societal costs. These costs will be combined with the QoL outcome measures (EQ-5D-5L), to calculate the incremental cost-effectiveness ratio of IMPACT compared to nonsurgical therapy in terms of cost per QALY over a 5-year time horizon.

### Plans to promote participant retention and complete follow-up {18b}

The patients will receive extensive information about the study set-up and requirements during the recruitment. The importance of completion of the follow-up will be stressed. Patients are allowed to stop at any time during the study and are not obliged to give a reason to discontinue. If possible, the patient will be asked to complete the online survey at 9 months after inclusion. Questionnaires are completed using an online survey, and therefore patients can do this at any convenient moment. All patients are reminded throughout the study to fill out the questionnaires during study visits. Throughout the follow-up period, the researchers will check responses and if necessary contact patients for completion of their follow-up.

### Data management {19}

Patient data will be collected with a GCP compliant eCRF (Castor EDC) [26]. Questionnaires will be answered online and output will be stored in SPSS. Back-ups in the study folder on the protected research server will be made regularly(once per 3 months). Informed consent and end-of-trial dates will be recorded in the electronic patient dossier and signed paper forms will be stored within our hospital in a locked room. (S)AEs will be recorded in the eCRF. To be able to reproduce study results and to help future users to understand and reuse data, all changes made to the raw data and all steps taken in the analysis will be documented in the eCRF and IBM SPSS (version 15.0.0.2, Chicago, IL). Source data will remain available in electronic patient record and OnlinePROMS. All research data, including patient material, will be archived for 30 years after the study has ended according to the guidelines for Advanced Therapy Medicinal Products (ATMPs).

### Confidentiality {27}

Research data will be stored using a study identification code for each participant. The key to the identification code list will only be available to the research team during the study and will be documented and safeguarded by the principal investigator according to research guidelines after completion of the study. No patient identification details will be reported in publications.

### Plans for collection, laboratory evaluation, and storage of biological specimens for genetic or molecular analysis in this trial/future use {33}

In the case of leftover material, this will be stored within the Cell Therapy Facility according to the ATMP legislation.

## Statistical methods

### Statistical methods for primary and secondary outcomes {20a}

Data will be analyzed using IBM SPSS (version 15.0.0.2, Chicago, IL). Data from the primary objective (KOOS, EQ-5D-5L) will be presented as continuous variables. To compare between the two treatment groups, a mixed model analysis will be performed, with treatment group and assessment date of the PROMs (e.g., baseline, 3 months, 6 months) as fixed factors. Differences will be considered statistically significant for the fixed effect of treatment groups if *p* < 0.05 [30]. The 95% confidence interval of the fixed effect size will be used to assess whether treatment difference reaches the minimally clinical important difference.

T1rho-scores will be calculated from the biochemical MRI scans; differences in T1rho-scores will be compared between the time points *t* = 0 (at inclusion before IMPACT or nonsurgical treatment) and *t* = 6 and 18 months after inclusion or surgery. Differences will be compared using Student’s *t* test. The differences in T1rho-score will be tested for normality using Q–Q plots. *P* values less than 0.05 will be considered significant. We will calculate the ICERs of IMPACT vs. nonsurgical treatment and vs. delayed surgical treatment in terms of cost/QALY over a 5-year time horizon. The effect of uncertainty in input parameters on the ICERs will be calculated by means of one-way sensitivity analysis as well as probabilistic sensitivity analyses using Monte Carlo simulation.

All (S)AE(I)s will be summarized and recorded including the nature, date and time of onset, date of resolution, determination of seriousness, severity, action taken, outcome, and possible causality to study treatment. SAE data will be presented in a descriptive manner.

### Interim analyses {21b}

There are no interim analyses planned.

### Methods for additional analyses (e.g., subgroup analyses) {20b}

There are no subgroup analyses planned.

### Methods in analysis to handle protocol non-adherence and any statistical methods to handle missing data {20c}

The primary outcome will be assessed using an intention-to-treat analysis. Missing data will be reduced to a minimum by using the appropriate measures described above. Mixed models do not require imputations for missing data. If any statistical method is needed to account for missing data in the secondary outcomes, multiple imputation will be used.

### Plans to give access to the full protocol, participant-level data, and statistical code {31c}

The datasets used and/or analyzed during the current study can be made available by the corresponding author upon reasonable request and in agreement with the research collaboration and data transfer guidelines of the UMC Utrecht.

## Oversight and monitoring

### Composition of the coordinating center and trial steering committee {5d}

This is a monocenter study designed, performed and coordinated in the UMC Utrecht. Day to day support for the trial is provided by:

Principle investigator: takes supervision of the trial and medical responsibility of the patients.

Data manager: organizes data capture, safeguards quality and data.

Study coordinator: trial registration, coordinates study visits, annual safety reports.

Study physician: identifies potential recruits, takes informed consent, ensures follow-up according to protocol.

The study team meets biweekly. There is no trial steering committee or stakeholder and public involvement group.

### Composition of the data monitoring committee, its role, and reporting structure {21a}

In agreement with the advice from the central Data Safety Monitoring Board (DSMB) committee of the UMC Utrecht, a DSMB has not been appointed for this study. The decision was based on the lack of SAEs in the phase I/II trial. Moreover, since this is not a blinded study, there is no DMSB required to protect blinding of the researchers and physicians. Lastly, due to the expected rapid inclusion and treatment of patients, interim assessments of a DSMB will not add value to the safety in this study. A rheumatologist knowledgeable in the field of allergic/immunologic reactions will be assigned as safety officer for this study. In case of SAEs, this safety officer will be contacted within 48 h. The safety officer will assess if the SAE is (definitely or possibly) related to treatment. In case of (possible) treatment relation, further safety measures will be taken on advice of the safety officer.

### Adverse event reporting and harms {22}

All adverse events reported by the subject or observed by the investigators will be recorded. The causality to the study treatment event will be recorded. Several complications are considered as AEs of Interest (AEIs) based on information from the previous trial and theory of the study procedures: arthralgia, swelling, or crepitation other/longer than may be expected and resulting in alteration in medical care, synovitis, surgical site infection, migration or dislocation of the graft, knee locking, hemarthrosis, arthrofibrosis, chondropathy (including a new cartilage lesion in the same knee or a secondary lesion), general surgery-related disorders (pneumonia, deep vein thrombosis, pulmonary embolism), disorders resulting from general or local anesthesia, and tissue hypertrophy. SAEs will be reported to the CCMO following the CCMO guidelines.

### Frequency and plans for auditing trial conduct {23}

An independent study monitor, Julius Clinical Research B.V (Zeist, The Netherlands), was appointed according to the guidelines of the Dutch Federation of University Medical Centers (NFU 2.0) (October 2012) for study-specific auditing. Based on these guidelines, the estimated risk for this study is considered moderate. The independent monitor makes two on-site visits per year and checks the presence and completeness of the investigation file. Moreover, the monitor checks the following data for 25% randomly picked patients: informed consents, inclusion and exclusion criteria, source data, and missing and reporting for (S)AEs/SUSARs. For more information, the monitoring plan can be consulted. Auditing can also take place by national or international health authorities, like the Dutch Health and Youth Care Inspectorate (IGJ).

### Plans for communicating important protocol amendments to relevant parties (e.g., trial participants, ethical committees) {25}

A “substantial amendment” is defined as an amendment to the terms of the CCMO application, or to the protocol or any other supporting documentation, that is likely to affect to a significant degree: the safety or physical or mental integrity of the subjects of the trial; the scientific value of the trial; the conduct or management of the trial; or the quality or safety of any intervention used in the trial.

All substantial amendments will be notified to the CCMO and to the competent authority. Non-substantial amendments will be recorded and filed. In case amendments concern or affect participants in any way, they are informed about the changes. If needed, additional consent will be requested and registered. Also, online trial registries will be updated accordingly.

### Dissemination plans {31a}

Results of this research will be disclosed completely in international peer-reviewed journals. Both positive and negative results will be reported. Patients will receive a laymen summary of the results in case they opted-in to receive outcomes on a study level.

## Discussion

This randomized controlled trial is designed to investigate efficacy of IMPACT compared to nonsurgical care. Safety of IMPACT one-stage surgery for articular cartilage defects will be monitored in 30 to 60 patients. Also, cost-effectiveness of IMPACT will be compared to nonsurgical care.

### Limitations

There are several limitations to consider. First, cartilage defects may lead to major disability with a high patient burden; therefore, patients are likely to request immediate surgical treatment. The possible delay of surgical treatment by allocation to the nonsurgical control group might impair patient inclusion or increase drop-out in the control group. Risk of drop-out will be minimized by properly informing the patients of the study set-up and goals. Secondly, we compare IMPACT surgery to nonsurgical therapy instead of ACI surgery. A nonsurgical control group was explicitly chosen due to the limited availability of ACI in Europe in the last decade. A comparison with ACI can be made retrospectively using our prospective registry, which includes data on safety, efficacy, and treatment and societal costs of the patients treated with ACI at our center. In addition, to the best of our knowledge, no randomized controlled trial has been performed previously that compares cell therapy to conservative treatment. Lastly, the follow-up period after surgery is relatively short (1.5 years) and long-term efficacy remains to be investigated. We ask permission of all patients to contact them after the study period in order to investigate long term follow-up and aim to include all patients in our prospective registry.

### Strengths

This clinical trial will provide insight into the efficacy of IMPACT compared to nonsurgical care. By the use of nonsurgical therapy as a comparator group, we will gain insight into the natural course of disease of nonsurgically treated patients. Moreover, we will establish a control group that can be used universally and independent of availability of (different types of) cell therapy for cartilage defects. Lastly, emergence of an immune response will be assessed by screening for anti-HLA antibodies in peripheral blood. This will provide useful insights in the in vivo behavior of MSCs, which can be transferred to other applications of MSCs, for example in regenerative medicine.

## Trial status

Recruiting started in July 2019. The current protocol is version 5 of 19-9-2019. Currently (15th of May 2020), we included ten patients. Patient recruitment is estimated to be completed around August 2021.

## Data Availability

The datasets used and/or analyzed during the current study will be made available from the corresponding author upon reasonable request.

## Abbreviations

ACI: Autologous chondrocyte implantation
ATMP: Advanced Therapy Medicinal Product
CCMO: Central Committee on Research Involving Human Subjects
DSMB: Data Safety Monitoring Board
eCRF: Electronic Case Report Form
EQ5D-5 L: EuroQol five dimensions five levels
GCP: Good Clinical Practice
GMP: Good Manufacturing Practice
IGJ: Health and Youth Care Inspectorate
IMPACT: Instant MSC Product accompanying Autologous Chondron Transplantation
KOOS: Knee Injury and Osteoarthritis Outcome Score
MSC: Multipotent mesenchymal stromal cell
NRS: Numeric Rating Scale
OA: Osteoarthritis
OOS: Out of specification
SAE: Serious adverse event
SUSAR: Suspected unexpected serious adverse reaction
WMO: Medical Research Involving Human Subjects Act

## References

[1] Buckwalter JA (1998). Articular cartilage: injuries and potential for healing. J Orthop Sports Phys Ther.

[2] Gelber AC, Hochberg MC, Mead LA, Wang N, Wigley FM, Klag MJ (2000). Joint injury in young adults and risk for subsequent knee and hip osteoarthritis. Ann Intern Med.

[3] Pisanu G, Cottino U, Rosso F, Blonna D, Marmotti AG, Bertolo C (2018). Large osteochondral allografts of the knee: surgical technique and indications. Joints.

[4] Brix M, Kaipel M, Kellner R, Schreiner M, Apprich S, Boszotta H (2016). Successful osteoconduction but limited cartilage tissue quality following osteochondral repair by a cell-free multilayered nano-composite scaffold at the knee. Int Orthop.

[5] Christensen BB, Foldager CB, Jensen J, Jensen NC, Lind M (2016). Poor osteochondral repair by a biomimetic collagen scaffold: 1- to 3-year clinical and radiological follow-up. Knee Surg Sport Traumatol Arthrosc.

[6] Brittberg M, Lindahl A, Nilsson A, Ohlsson C, Isaksson O, Peterson L (1994). Treatment of deep cartilage defects in the knee with autologous chondrocyte transplantation. New Engl J Med.

[7] Foldager CB, Gomoll AH, Lind M, Spector M (2012). Cell seeding densities in autologous chondrocyte implantation techniques for cartilage repair. Cartilage.

[8] Grande DA, Pitman MI, Peterson L, Menche D, Klein M (1989). The repair of experimentally produced defects in rabbit articular cartilage by autologous chondrocyte transplantation. J Orthop Res.

[9] Knutsen G, Drogset JO, Engebretsen L, Grøntvedt T, Isaksen V, Ludvigsen TC (2007). A randomized trial comparing autologous chondrocyte implantation with microfracture: findings at five years. J Bone Joint Surg Am.

[10] Saris DBF, Vanlauwe J, Victor J, Almqvist KF, Verdonk R, Bellemans J (2009). Treatment of symptomatic cartilage defects of the knee: characterized chondrocyte implantation results in better clinical outcome at 36 months in a randomized trial compared to microfracture. Am J Sports Med.

[11] Marlovits S, Hombauer M, Truppe M, Vècsei V, Schlegel W (2004). Changes in the ratio of type-I and type-II collagen expression during monolayer culture of human chondrocytes. J Bone Joint Surg Br.

[12] Schnabel M, Marlovits S, Eckhoff G, Fichtel I, Gotzen L, Vécsei V (2002). Dedifferentiation-associated changes in morphology and gene expression in primary human articular chondrocytes in cell culture. Osteoarthr Cartil.

[13] de Windt TS, Sorel JC, Vonk LA, Kip MMA, Ijzerman MJ, Saris DBF (2017). Early health economic modelling of single-stage cartilage repair. Guiding implementation of technologies in regenerative medicine. J Tissue Eng Regen Med.

[14] EMA. ChondroCelect 2020 https://www.ema.europa.eu/en/medicines/human/EPAR/chondrocelect. Accessed 15 Sep 2020.

[15] EMA. Closure of EU manufacturing site for MACI. 2014. https://www.ema.europa.eu/en/documents/referral/maci-article-20-procedure-closure-eu-manufacturing-site-maci_en.pdf. Accessed 3 Apr 2020.

[16] Chen WH, Lai MT, Wu ATH, Wu CC, Gelovani JG, Lin CT (2009). In vitro stage-specific chondrogenesis of mesenchymal stem cells committed to chondrocytes. Arthritis Rheum.

[17] Tsuchiya K, Chen G, Ushida T, Matsuno T, Tateishi T (2004). The effect of coculture of chondrocytes with mesenchymal stem cells on their cartilaginous phenotype in vitro. Mater Sci Eng C.

[18] Mo XT, Guo SC, Xie HQ, Deng L, Zhi W, Xiang Z (2009). Variations in the ratios of co-cultured mesenchymal stem cells and chondrocytes regulate the expression of cartilaginous and osseous phenotype in alginate constructs. Bone.

[19] Bekkers JEJ, Tsuchida AI, van Rijen MHP, Vonk LA, Dhert WJA, Creemers LB (2013). Single-stage cell-based cartilage regeneration using a combination of Chondrons and Mesenchymal stromal cells. Am J Sports Med.

[20] de Windt TS, Hendriks JA, Zhao X, Vonk LA, Creemers LB, Dhert WJA (2014). Concise review: unraveling stem cell cocultures in regenerative medicine: which cell interactions steer cartilage regeneration and how?. Stem Cells Transl Med.

[21] de Windt TS, Vonk LA, Slaper-Cortenbach ICM, Nizak R, van Rijen MHP, Saris DBF (2017). Allogeneic MSCs and recycled autologous chondrons mixed in a one-stage cartilage cell transplantion: a first-in-man trial in 35 patients. Stem Cells.

[22] de Windt TS, Vonk LA, Slaper-Cortenbach ICM, van den Broek MPH, Nizak R, van Rijen MHP (2017). Allogeneic Mesenchymal stem cells stimulate cartilage regeneration and are safe for single-stage cartilage repair in humans upon mixture with recycled autologous chondrons. Stem Cells.

[23] Roos E (2020). KOOS FAQ.

[24] Muller KE, Edwards LJ, Simpson SL, Taylor DJ (2007). Statistical tests with accurate size and power for balanced linear mixed models. Statist Med.

[25] SAS Institute (2014). Power and sample size for MANOVA and repeated measures with the GLMPOWER procedure.

[26] Castor EDC (2020). Castor Electronic Data Capture.

[27] Institute for Medical Technology Assessment. Questionnaires. 2020. https://www.imta.nl/questionnaires/ Accessed 13 May 2020.

[28] Dutch Healthcare Authority. 2020. https://zorgproducten.nza.nl/. Accessed 14 Sep 2020.

[29] Zorginstituut Nederland (2016). Richtlijn voor het uitvoeren van economische evaluaties in de gezondheidszorg.

[30] Seltman HJ (2018). Mixed Models. Experimental design and analysis.

